# Machine Learning-Enabled Image Comparability Assessment for Flow Imaging Microscopy Across Platforms

**DOI:** 10.3390/ph19010107

**Published:** 2026-01-07

**Authors:** Zhenhao Zhou, Sha Guo, Youli Tian, Hanhan Li, Zhiyun Qi, Xiaoying Chen, Jiaxin Li, Dongjiao Li, Pengfei He, Hao Wu

**Affiliations:** 1 School of Pharmaceutical Engineering, Shenyang Pharmaceutical University, Shenyang 110016, China; zzh13793871380@163.com (Z.Z.); chem_hannah@163.com (H.L.); qizhiyun@nifdc.org.cn (Z.Q.); 13104146783@163.com (X.C.); 13840646328@163.com (J.L.); 15140538506@163.com (D.L.); 2 WHO Collaborating Centre for Standardization and Evaluation of Biologicals, State Key Laboratory of Drug Regulatory Science, NHC Key Laboratory of Research on Quality and Standardization of Biotech Products, NMPA Key Laboratory for Quality Research and Evaluation of Biological Products, National Institutes for Food and Drug Control, Beijing 102629, China; guosha@nifdc.org.cn; 3 School of Automation and Intelligence Sensing, Shanghai Jiao Tong University, Shanghai 200240, China; s1030512149@sjtu.edu.cn

**Keywords:** injectable biopharmaceuticals, machine learning, standardization study, subvisible particles, flow imaging microscopy (FIM)

## Abstract

**Background/Objectives**: The rapid development of biopharmaceuticals has heightened attention from both industry and regulatory agencies toward product quality, particularly regarding subvisible particles as a critical quality attribute. Existing pharmacopoeial methods, Light Obscuration (LO) and Microscopic Particle Count (MC), exhibit limitations in meeting increasingly refined analytical requirements. Flow Imaging Microscopy (FIM) technology shows promise as an alternative, yet its standardized methodologies are still under development. **Methods**: This study employed polystyrene microsphere standard beads and intravenous immunoglobulin to perform instrument standardization and consistency evaluations on FIM instruments sharing the same operating principles but from different manufacturers. The consistency and transferability of particle counting across platforms were assessed. Additionally, particle images obtained from parallel testing on two platforms were classified using confusion matrices based on convolutional neural networks and the Unified Manifold Approximation and Projection (UMAP) dimensionality reduction method. **Results**: This study investigated the consistency and developed a transfer strategy for particle counting results across different FIM platforms. Analysis of particle image classification confirmed the consistency of image-based categorization while also revealing the complexity associated with cross-platform image recognition. **Conclusions**: The findings provide valuable insights for the further standardization of Flow Imaging Microscopy, supporting its potential as a reliable analytical tool for subvisible particle analysis in biopharmaceutical quality control.

## 1. Introduction

The rapid development of injectable biopharmaceuticals is reshaping the modern medical landscape [1,2]. These biopharmaceuticals, including monoclonal antibodies, recombinant proteins, vaccines, and cell therapy products, have become the cornerstones in the treatment of malignant tumors, autoimmune diseases, and genetic disorders [3,4,5]. However, the formation of subvisible particles (SVPs, 2–100 μm in diameter) by these structurally complex drugs during manufacturing, storage, transportation and clinical use [6,7,8] poses significant clinical safety risks [9]. The administration of formulations containing such particles via intravenous, subcutaneous, or intramuscular injections induces clinical adverse reactions, the nature of these reactions is largely determined by particle size and material [10,11]. Particles in the 10–25 μm range may clog pulmonary capillaries and cause acute respiratory distress [12], while protein aggregates smaller than 10 μm may activate the complement system and trigger systemic inflammatory response [13,14,15]. Glass flakes (e.g., from vial containers) or rubber particles larger than 50 μm may be trapped in the liver sinusoids or glomeruli, leading to local granuloma formation and organ dysfunction [16]. More critically, antibody aggregates or protein–particle complexes possess strong unwanted immune response [17], potentially inducing the production of neutralizing antibodies [18], which not only reduce drug efficacy but may also trigger life-threatening allergic reactions [19,20]. For example, Omontys^®^ was withdrew from the market within a year due to severe allergic reactions, and nanoparticle tracking analysis and flow imaging of subvisible particles revealed that insoluble particulate concentrations were significantly higher in multi-dose vials associated with hypersensitivity reactions [21]. Several national regulatory authorities have recalled monoclonal antibody drugs due to particle contamination, highlighting the urgency of this issue [22,23,24]. According to a summary by Martin et al. of 89 approved antibody-based biotherapeutics up to 2020, intravenous administration was the most common route (59 products, 66.2%), followed by subcutaneous administration (27 products, 30.4%) [25]. Therefore, establishing a particle detection system capable of precisely quantifying, classifying by size, and identifying material composition has become a critical need for ensuring patient safety, optimizing manufacturing processes, and meeting global regulatory requirements [26].

The subvisible particle detection methods mandated by various pharmacopoeias, including the Chinese Pharmacopoeia (ChP 0903, 2020), the United States Pharmacopeia (USP <788>), and the European Pharmacopoeia (EP 2.9.19), primarily rely on Light Obscuration (LO) and Microscopic Particle Count (MC) methods [27]. In addition, the revised USP <1788> has introduced flow imaging microscopy as an orthogonal method to supplement these compendial approaches for subvisible particle characterization. The LO method estimates particle size and quantity by measuring the attenuation of light intensity as particles pass through the detection area. While it offers the advantage of automation, it is limited in its inability to distinguish between translucent protein aggregates and opaque silicone oil droplets [28], and may have limitations in accurately sizing non-spherical particles [29]. Additionally, variations in the refractive index between the sample and the surrounding medium may lead to counting biases [30]. The MC method, while providing morphological information, is heavily reliant on manual inspection, which introduces subjectivity and leads to poor reproducibility. Operators must manually identify and count particles on a 0.45 μm filter membrane, resulting in considerable time consumption for a single sample and a low throughput that cannot meet the demand for batch testing of biopharmaceuticals. Moreover, inter-laboratory variations due to subjective differences in operator judgment may lead to increased coefficient of variation in particle counts, limiting the reproducibility and comparability of the data [31]. This issue is particularly pronounced for biopharmaceutical therapeutics, where the limitations of both methods in sensitivity, specificity, and traceability highlight the urgent need for the development of next-generation detection platforms capable of acquiring higher-dimensional information.

Flow Imaging Microscopy (FIM) technology utilizes a precise fluid control system to create laminar flow within a transparent microchannel, combined with a high-speed CMOS camera and pulsed stroboscopic illumination system to achieve dynamic in situ imaging and digital capture of individual particles [32]. Its core technological advantage lies in pixel-based absolute particle size measurement (with a resolution of up to 0.5 μm), eliminating the optical property dependence of the LO method. Through a particle classification model built on morphological parameters (such as circularity, aspect ratio, and transparency), FIM can accurately distinguish silicone oil droplets (high sphericity, low transparency), protein aggregates (irregular shape, medium transparency), and fibers (high aspect ratio) [33]. Additionally, FIM establishes a full-field image database, providing visual evidence for particle source tracing and the study of particle formation processes [26,33]. Studies have shown that FIM achieves higher counting accuracy than LO in counting accuracy for particles larger than 10 μm and is capable of detecting sub-10 μm translucent particles that traditional methods may overlook [28,34]. This combination of high sensitivity, objective quantification, and intelligent classification makes FIM an ideal tool for the characterization of particles in biopharmaceuticals, particularly in applications such as stability studies, packaging compatibility evaluations and manufacturing process troubleshooting. Consistent with its current regulatory positioning in USP <1788>, FIM is primarily applied as an orthogonal technique to complement compendial particle counting methods by providing additional morphological and mechanistic insights.

Flow Imaging Microscopy technology has demonstrated significant advantages; however, its standardized application in global quality control systems still faces critical challenges [35]. Specifically, achieving consistent and accurate particle counting across platforms is not sufficient without first ensuring image consistency, where images of the same sample obtained from different instruments are recognized as identical. Only after confirming this image consistency can particle counts and other measurements be reliably calibrated and compared across different platforms. Few studies have been conducted to validate both the consistency of detection results and the transferability of data across different instrument platforms [36]. This directly impacts the compliance of multinational pharmaceutical companies in achieving data mutual recognition across multiple sites and the recognition of method reliability by regulatory agencies. This study aims to provide valuable insights through multi-dimensional image analysis to address this issue. Firstly, a gradient dilution series of polystyrene standard microspheres, certified by NIST (National Institute of Standards and Technology) traceability, was used for cross-calibration of two FIM systems from different manufacturers to assess their counting linearity and particle size deviation in detecting ideal spherical particles. Secondly, typical therapeutic protein injectable formulations were selected to prepare low and high particle concentration gradient samples. These samples were analyzed for data reproducibility in complex biological matrices through parallel detection with two instruments. Finally, machine learning-driven image comparability analysis was introduced by extracting features from millions of particle images captured by both systems. A convolutional neural network (CNN) classification model was constructed, and the consistency of morphological recognition of homologous particle populations by the two instruments was validated through confusion matrices and UMAP dimensionality reduction visualization. This comprehensive study not only confirms the robustness of FIM across platforms but also provides experimental insights for establishing unified standards for biopharmaceutical particle detection.

## 2. Results

### 2.1. Analysis of Particle Size and Concentration Results

**Figure 1:**
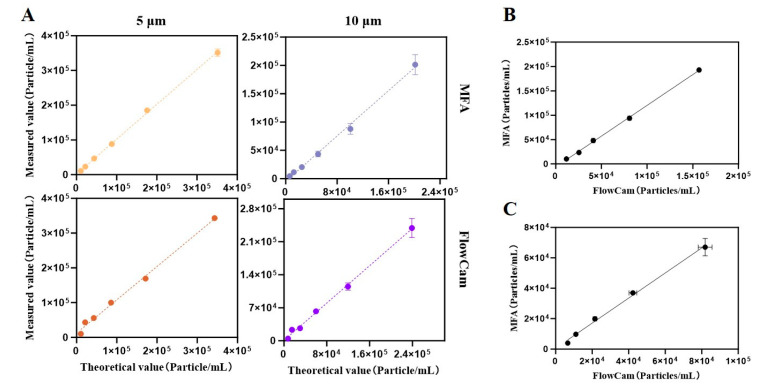
Measurement of different sizes polystyrene standard beads by MFA and FlowCam: (**A**) Linearity of measurement; (**B**) Correlation and consistency in the detection of 5 μm standard beads and (**C**) 10 μm standard beads between two platforms. In this figure, dashed or solid lines represent linear fits. The bars denote error bars; where bars are not visible, the error is smaller than the marker size.

For the gradient dilutions of polystyrene microspheres, both instruments exhibited excellent linearity (R^2^ ≥ 0.98, Figure 1A). In both cases, the R^2^ values for the nominal 5 μm microspheres were higher than those for the 10 μm microspheres. Notably, MFA achieved superior linearity for the 5 μm particles compared with FlowCam. Specifically, MFA yielded a linear regression equation of Y = 1.003X + 2010 with an R^2^ of 0.9992 for 5 μm microspheres, and Y = 1.001X − 4617 with an R^2^ of 0.9857 for 10 μm microspheres. For FlowCam, the regression equations were Y = 0.9577X + 12583 with an R^2^ of 0.9945 for 5 μm microspheres, and Y = 0.9897X + 860.4 with an R^2^ of 0.9887 for 10 μm microspheres.

It is noteworthy that MFA demonstrated slightly better linearity for 5 μm microspheres (R^2^ = 0.9992) compared with FlowCam (R^2^ = 0.9945), which may be attributed to subtle differences in fluidic control between the two platforms. MFA operated at a lower flow rate (0.12 mL/min), which prolonged imaging time for individual particles but was associated with fewer frames per second. In contrast, FlowCam employed a higher flow rate (0.15 mL/min), which increased throughput and frame capture, but may have introduced minor variability. Despite these differences, both instruments achieved R^2^ values greater than 0.985 for the critical particle size range (larger than or equal to 10 μm), with regression slopes close to 1 (Table 1). These findings confirmed the cross-platform robustness of both MFA and FlowCam in quantifying spherical particles. The consistency of the two instruments in detecting the same standard bead samples was shown in Figure 1B,C. For the detection of 5 μm standard beads, the linear regression equation for the results obtained by both instruments was Y = 1.263X − 6055, with an R^2^ value of 0.9991. For the 10 μm standard beads, the linear regression equation was Y = 0.8241X + 597.5, with an R^2^ value of 0.9866. The detection of standard beads demonstrated acceptable consistency between the two instruments.

### 2.2. Measurement of Particles Generated from Protein Formulation

**Figure 2:**
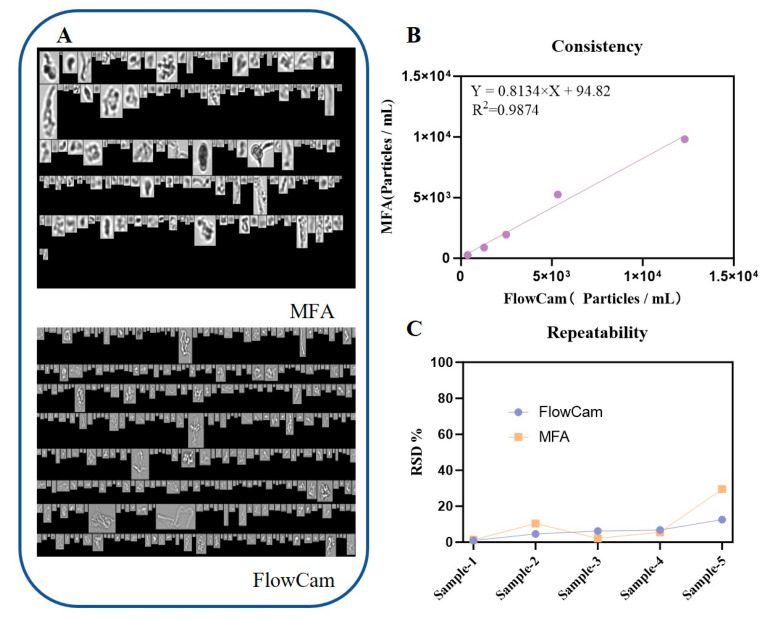
Results of subvisible particle analysis of different biological samples. (**A**) Representative particle images captured by both instruments; (**B**) Cross-platform consistency between MFA and FlowCam in particle quantification; (**C**) Repeatability of MFA and FlowCam measurements on the same sample.

Both instruments completed three replicate measurements of the same sample within 10 min, and the relative standard deviations (RSD) of total particle counts remained consistently low. For five randomly selected stressed IVIG samples at different concentrations, the results obtained from MFA and FlowCam showed strong agreement. A linear regression of particle counts between the two platforms yielded Y = 0.8134X + 94.82 with an R^2^ value of 0.9874 (Figure 2B), confirming the reproducibility of measurements across instruments.

**Figure 3:**
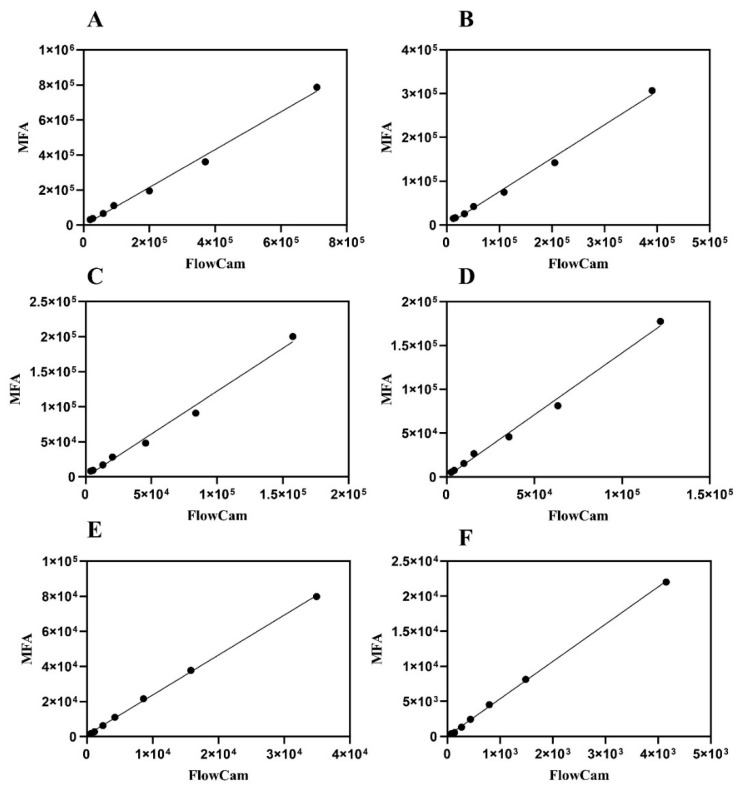
Comparison of particle size distributions (2–100 μm) in gradient-diluted stressed IVIG samples analyzed by MFA and FlowCam. (**A**) 2–100 μm; (**B**) 2–5 μm; (**C**) 5–10 μm; (**D**) 10–25 μm; (**E**) 25–50 μm; (**F**) 50–100 μm.

To quantitatively assess the relationship between the particle concentrations measured by the two platforms across different size populations, linear regression analysis was employed. This approach provides a metric for correlation and systematically reveals any concentration-dependent biases between the instruments, even if the underlying particle detection physics within a given size range may be complex. The consistency of MFA and FlowCam in analyzing gradient-diluted stressed IVIG samples is illustrated in Figure 3. Across the particle size distribution range of 2–100 μm, both platforms demonstrated a high degree of concordance. Linear regression of total particle concentrations yielded Y = 1.079X − 1230 with an R^2^ value of 0.9940. For subvisible particles below 25 μm, the slopes of the linear regression equations between the two instruments ranged from 0.7 to 1.5, indicating an acceptable level of agreement between two instruments (Table 2). These high R^2^ values indicate a strong linear relationship between the instruments across size ranges; however, the slopes deviating from unity, especially for larger particles, confirm the presence of systematic biases that would require calibration for achieving quantitative agreement. A Bland–Altman analysis of agreement for these measurements is provided in the Supplementary Materials (Figures S1 and S2), which visually corroborates these systematic biases and their dependence on particle size and concentration.

Overall, particle concentrations measured by the MFA were higher than those obtained by the FlowCam across most size ranges, except for the 2 to 5 μm fraction. The discrepancy became more pronounced for larger particles, particularly for particles above 50 μm, where the slope of the linear regression exceeded 5. This difference might be attributed to the lower flow rate used in MFA (0.12 mL/min), which stabilized the motion of large particles, whereas the higher flow rate of FlowCam (0.15 mL/min) may destabilize particle trajectories. Furthermore, although FlowCam operated at a high frame rate, the imaging of rapidly moving large particles remained susceptible to motion blur due to their significant displacement within the finite exposure time of each frame. This blur could reduce the effective resolution, potentially leading to an underestimation of particle counts, especially for those with indistinct edges. Conversely, for samples with relatively low particle numbers, adsorption of protein aggregates onto the flow cell might become more influential, resulting in lower counts in MFA compared with FlowCam, as observed in Figure 2B.

Despite these differences, the linear regression R^2^ values for both instruments consistently exceeded 0.99, indicating that the discrepancies in particle counts between MFA and FlowCam primarily arise from inherent system-level biases. These findings suggested that calibration and hardware optimization could provide a feasible pathway toward achieving cross-platform harmonization in particle quantification [34].

### 2.3. Clustering and Parameter Analysis of Images Using Machine Learning

**Figure 4:**
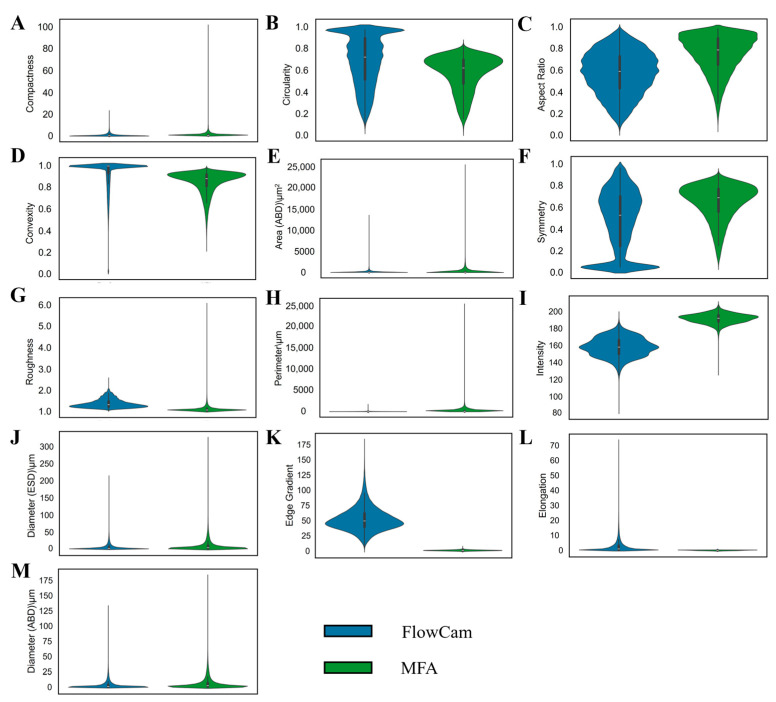
Statistical distributions of proteinaceous particle parameters measured by the two instruments, including (**A**) Compactness; (**B**) Circularity; (**C**) Aspect Ratio; (**D**) Convexity; (**E**) Area (ABD (Area-Based Diameter)); (**F**) Symmetry; (**G**) Roughness; (**H**) Perimeter; (**I**) Intensity; (**J**) Diameter (ESD (Equivalent Spherical Diameter)); (**K**) Edge Gradient; (**L**) Elongation and (**M**) Diameter (ABD). Please refer to Table S1 for the definitions of the key characteristic parameters.

Figure 4 presented violin plots comparing the statistical distributions of thirteen morphological parameters of proteinaceous particles measured by FlowCam and MFA. Notably, the two instruments exhibited varying degrees of distributional divergence across most parameters. These differences were particularly pronounced for Intensity (Figure 4I) and Edge Gradient (Figure 4K), where both the distributional shapes and central tendencies showed clear shifts between platforms. Such systematic discrepancies in measurement characteristics indicated that directly pooling raw data from the two instruments would introduce substantial analytical bias.

**Figure 5:**
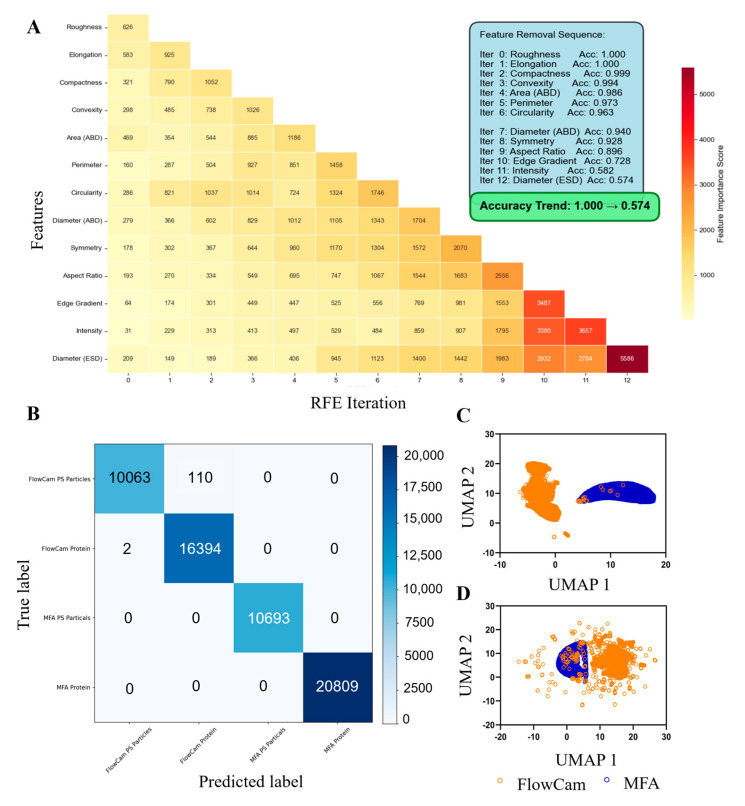
Machine learning and morphological comparison of protein particle images acquired by the two flow imaging platforms. (**A**) Feature-importance trajectory derived from XGBoost-based recursive feature elimination; (**B**) Confusion matrices illustrating cross-platform classification performance; (**C**) UMAP projections highlighting instrument-dependent clustering of protein particle features; (**D**) UMAP projections after excluding Intensity and Edge Gradient, demonstrating their dominant contribution to inter-instrument feature divergence.

To quantitatively assess the functional implications of the observed parameter differences, we trained an XGBoost classifier and applied a recursive feature elimination (RFE) approach to deconstruct its predictive performance. This strategy enabled us to rank the relative importance of the 13 morphological parameters based on their contributions to accurate classification of proteinaceous particles, rather than relying solely on their statistical variance. The RFE analysis (Figure 5A) revealed a distinct hierarchy of feature importance. Parameters such as Diameter (ESD), Intensity, and Edge Gradient emerged as the most robust predictors; even after the removal of a substantial number of other parameters, the model maintained high accuracy (>0.89). In contrast, parameters including Roughness, Elongation, and Compactness were identified as having minimal functional impact, as their early elimination during the initial RFE iterations resulted in only negligible reductions in model performance (accuracy decreasing marginally from 1.000 to 0.999). Notably, the feature importance ranking derived from the RFE analysis aligned well with the pronounced differences observed in Intensity and Edge Gradient between the two instruments in Figure 4.

A classification analysis of polystyrene microsphere and protein particle images captured by the two instruments was conducted using confusion matrices, as shown in Figure 5B. Model performance metrics, including precision, recall and F1 score were summarized in Table 3. Nearly all particle images were correctly classified, with only a very small fraction of polystyrene microspheres misclassified as protein particles. Overall, both MFA and FlowCam demonstrated excellent classification performance across different particle types. However, for images of the same particle type, the results indicated that the two instruments may not yet achieve full cross-recognition. For example, protein particle images captured by FlowCam and MFA were completely separated into two distinct categories by the confusion matrix, with a classification accuracy of 100%. This observation was further supported by feature mapping using UMAP (Figure 5C), where protein particle images from the two platforms showed minimal overlap. Comparative dimensionality reduction analysis of particle feature distributions using PCA and t-SNE had also supported this conclusion, as detailed in Figure S3. This discrepancy can be attributed to inherent differences in the hardware configurations of the two systems. The MFA system operated with a higher background brightness and a native 4K image resolution, while the FlowCam system is configured for 1080P resolution with a corresponding illumination intensity. These parameter differences directly affected image detail and contrast, which might lead to variations in particle characterization results.

In contrast, marked differences were observed in Intensity and Edge Gradient parameters. Intensity refers to the average grayscale value of pixels composing a particle, calculated as the total grayscale value divided by the number of pixels. Edge Gradient describes the average intensity of pixels at the particle boundary; higher values indicate sharper edges, whereas lower values reflect blurred boundaries. These parameters are influenced by factors such as depth of field, illumination, and image clarity. When Intensity and Edge Gradient were excluded, UMAP-based feature mapping revealed increased overlap of protein particle images between MFA and FlowCam (Figure 5D), suggesting that inherent hardware-dependent differences may significantly impact cross-platform recognition of particle images.

### 2.4. Classification of Particles in Different Platforms

**Figure 6:**
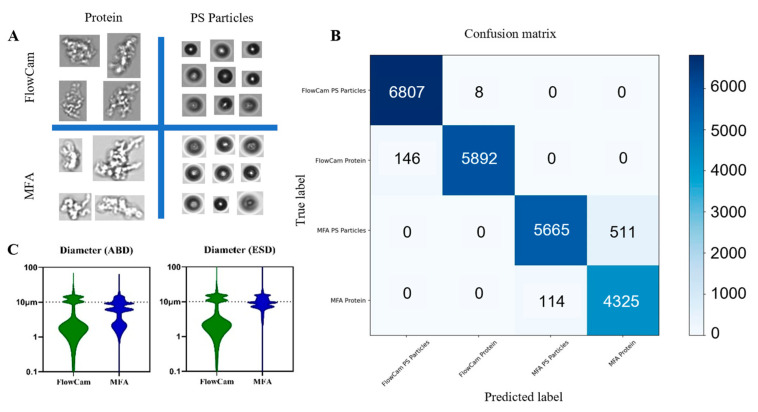
Detection results of mixed particle samples. (**A**) Particle images captured by MFA and FlowCam; (**B**) Validation of model generalization using confusion matrix; (**C**) Examination of particle parameters during the detection of mixed particle samples by MFA and FlowCam.

The general morphology of protein aggregates and polystyrene beads captured by FlowCam and MFA in mixed-particle samples is shown in Figure 6A. As expected, the two particle types exhibited pronounced morphological differences, providing a clear visual basis for manual particle classification by trained analysts. Manual review of individual particle images from single runs revealed that FlowCam captured 6038 images of protein aggregates and 6815 images of polystyrene beads, with protein particles accounting for 46.98% of all detected particles. In contrast, MFA captured 4439 images of protein aggregates and 6176 images of polystyrene beads, corresponding to 41.82% protein particles. The difference of approximately 5% between instruments indicated a reasonable level of agreement in mixed-particle classification. Figure 6C depicted the feature distribution patterns for mixed-particle samples, with a prominent peak near 10 μm, reflecting consistent detection tendencies toward polystyrene calibration beads across instruments. This further supported the feasibility and general applicability of using polystyrene beads for preliminary instrument calibration.

When the machine-learning clustering model developed in Section 2.3 was applied to classify mixed-sample particles, the confusion-matrix outputs closely aligned with manual classification results. Specifically, protein particles accounted for 46.40% and 43.29% of classified images in the FlowCam and MFA datasets, respectively. These findings demonstrated the strong generalization capability of the model when applied to heterogeneous particle populations not identical to the training set, offering empirical support for future development of offline, multi-platform particle classification and traceability frameworks. The precision, recall, and F1-scores of the trained models are summarized in Table 4.

## 3. Discussion

In this study, two flow imaging microscopy (FIM)-based instruments were employed to analyze regular-shaped polystyrene standards, randomly selected biological formulations, and gradient-diluted high particle concentration samples, with the goal of assessing the feasibility and robustness of FIM as a particle characterization method for injectable biologics. Compared with microscopic particle counting, which suffers from poor intra- and inter-laboratory consistency and inaccurate size estimation due to operator-dependent subjectivity, FIM achieved low relative standard deviations (less than or equal to 10%) through automated and intelligent detection workflows, effectively minimizing human bias. For stress-induced high-concentration heterogeneous particle populations, both instruments demonstrated strong correlation across size distribution ranges. Notably, both MFA and FlowCam adopt ESD-based algorithms for particle sizing, and the harmonization of this algorithm strengthened resistance to matrix interference, thereby ensuring high consistency in particle counts and morphometric features. Furthermore, machine learning analysis confirmed the strong classification capability of FIM across different particle types, while also indicating that cross-platform recognition of identical particle images was strongly influenced by hardware-specific differences, suggesting that certain image parameters may need to be selectively normalized during standardization studies.

The clinical value of FIM lies primarily in its ability to accurately identify high-risk particle populations. The demonstrated consistency of MFA and FlowCam in detecting 10–25 μm particles (R^2^ = 0.9936) was of particular significance, as this size range corresponded to the “high-risk window” for pulmonary capillary occlusion by silicone oil droplets and complement activation by protein aggregates [12,19,37]. This range represented particles that could cause significant immunogenic responses, making precise identification crucial for patient safety. Furthermore, the morphology-based classification of FIM (e.g., by circularity and transparency) enhanced its ability to distinguish between different particle types, with silicone oil droplets exhibiting high sphericity (circularity > 0.9) and low transparency, while protein aggregates displayed irregular shapes and semi-transparency [33]. This material-specific characteristic was beyond the capability of light obscuration methods, which could not effectively differentiate between particle types. Finally, the excellent cross-platform agreement of FIM for larger particles (≥25 μm, R^2^ = 0.9993) ensured compliance with stringent pharmacopoeial requirements, further strengthening its role in biopharmaceutical quality control. Additionally, the image database generated by FIM enabled tracking of aggregate formation dynamics, providing early warnings of immunogenicity risks and helping to prevent recalls due to particulate contamination.

A significant advantage of FIM over conventional methods is its ability to capture semi-transparent protein aggregates in the 2–10 μm range, which are often overlooked by conventional methods. Although these particles are not currently subject to mandatory pharmacopoeial monitoring, they may adsorb complement factors and trigger inflammatory responses [20]. By accurately detecting and quantifying these subvisible particles, FIM provides critical data that can help mitigate such “hidden risks” in biopharmaceutical formulations. Furthermore, the morphological analysis capability of FIM enables it to directly link particle characteristics to specific manufacturing failures. For example, highly spherical silicone oil droplets may arise from excessive syringe siliconization or rapid filling speeds [38], while irregular protein aggregates may indicate stress-induced instability or excipient incompatibility [2,39,40], elongated fibers may originate from filter shedding or container delamination; and opaque black rubber particles may be associated with stopper abrasion [41]. By capturing full-field images, FIM establishes a traceable chain of visual evidence for regulatory submissions, for example, by linking silicone oil droplet profiles to filling line faults or validating particle distribution changes following process modifications.

This study confirmed that FIM delivers comparable quantitative results (e.g., particle counts and size distributions) across platforms when using a unified analysis algorithm, demonstrating a key aspect of methodological robustness. Furthermore, the application of machine learning not only validated its classification capabilities but also explicitly highlighted the current limitations in cross-platform image recognition due to hardware-based feature discrepancies, thereby providing a clear rationale and direction for future standardization work. Future algorithmic advancements may enable cross-recognition and classification of particle images across platforms. However, several challenges remain before full standardization can be achieved. From a technical perspective, the particles (smaller than 2 μm) imposed by optical diffraction limits may necessitate the integration of nanoparticle tracking analysis (NTA) for complete size coverage. Even with harmonized algorithms, variations in fluid dynamics within flow cells may bias particle counts. Ideally, stable laminar flow should ensure uniform capture frequencies across the entire field of view; however, in practice, particles near the cell edges may traverse less efficiently than those at the center, or conversely may be captured redundantly, necessitating zoned acquisition or de-duplication strategies. In addition, elevated shear forces may shed protein aggregates adhering to flow cell surfaces, introducing counting errors and underscoring the need for low-shear sample introduction systems. Finally, although different FIM instruments employ similar detection principles for subvisible particles, variations in key hardware components—such as flow cell design, camera specifications, and light source characteristics—result in significant disparities in acquired particle image quality. For instance, in this study, the two instruments utilized cameras with different depths of field. Consequently, identical particles appeared with different measured sizes due to variations in edge gradient caused by the optical disparity [42]. These inherent hardware differences can induce systematic biases in particle sizing and quantification through algorithmic processing, underscoring the critical need for cross-platform data normalization and hardware standardization.

**Figure 7:**
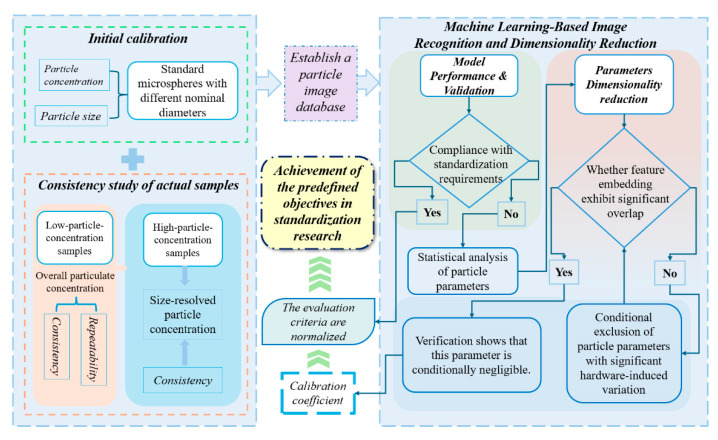
Proposed framework for the standardization of flow imaging microscopy (FIM) in subvisible particle analysis of injectable biopharmaceuticals.

As illustrated in Figure 7, the proposed framework outlines a strategy for the standardization of FIM in particle analysis of injectable biologics, offering guidance for future studies aimed at establishing harmonized protocols for inter-platform recognition and evaluation. Inter-instrument calibration and data harmonization across platforms often require coordinated implementation by regulatory authorities. Therefore, while the proposed framework provides a feasible and pragmatic approach for cross-platform data interpretation, it is not intended as a universally applicable standard and may require adaptation to accommodate different regulatory environments and quality control practices in other regions. Leveraging its high sensitivity, objectivity, and intelligent classification capabilities, FIM is well positioned not only to meet increasingly stringent pharmacopoeial requirements but also to drive a paradigm shift in biopharmaceutical quality management—from end-point quality control to proactive risk mitigation at the source. While the machine learning framework presented provides diagnostic insights for standardization, its practical integration into routine quality control would benefit from a focus on standardizing the underlying feature extraction and calibration steps, thereby reducing the need for complex model deployment in daily operations. Furthermore, for reliable deployment in regulated environments, any future implementation would require a model lifecycle management plan to address risks such as model drift, incorporating continuous performance monitoring, periodic re-validation, and maintained expert oversight. The framework presented here serves first as a diagnostic research tool to identify barriers in harmonization. The insights it generates are intended to pave the way for its future application as an enabling PAT tool and, ultimately, contribute to the evolution of more robust and standardized release methodologies for subvisible particle analysis. Ultimately, FIM provides a powerful technical foundation for ensuring patient safety and supporting lean manufacturing in biopharmaceutical production.

## 4. Materials and Methods

### 4.1. Materials

Polystyrene microspheres with nominal diameters of 5 μm and 10 μm in deionized water suspension were obtained from Thermo Fisher Scientific (Waltham, MA, USA). Water for injection was provided by Cisen Pharmaceutical Ltd. (Jining, China) and used as the diluent. Intravenous human immunoglobulin (IVIG, 2.5 g/50 mL per vial) was supplied by Shandong Taibang Biopharmaceutical Co., Ltd. (Tai’an, China) and diluted with saline from Liaoning Minkan Pharmaceutical Co., Ltd. (Dalian, China). PES water-based membranes with a pore size of 0.22 μm, polypropylene (PP) centrifuge tubes (15 mL and 50 mL), and 10 mL sterile syringes were purchased from Shenyang Yuwang Huabo Instrument Co., Ltd. (Shenyang, China).

IVIG was selected as a representative model particle for antibody-based injectables in this study. This choice was based on its morphological characteristics, which are broadly representative of particles found across various therapeutic protein formulations, including monoclonal antibodies (mAbs) and antibody–drug conjugates (ADCs), as supported by preliminary characterization (see Figure S4).

### 4.2. Testing System Setup

FlowCam 8100 (Yokogawa Fluid Imaging Technologies, Tokyo, Japan) was equipped with a flow cell that had an 80 μm field of view (FOV). Tergazyme protease (Sigma-Aldrich, St. Louis, MO, USA) was used as the cleaning solution. Prior to each measurement, 1 mL of cleaning solution was introduced into the flow cell at 6 mL/min, followed by deionized water filtered through a double-layer 0.22 μm PES membrane at 6 mL/min to remove residual particles. For the measurement to be valid, the concentration of subvisible particles in the filtered water must not exceed 200 particles/mL. For measurement, 200 μL sample was carefully aspirated with a pipette and slowly loaded into the sample chamber to avoid bubbles. The sample was passed through the flow cell at 0.15 mL/min at room temperature, with image acquisition at 27 frames per second. Particles larger than or equal to 2 μm were recorded and analyzed.

Microflow Analyzer (MFA, Dabiqing Biotechnology Co., Ltd., Suzhou, China): The flow cell was quartz, 1000 μm wide and 100 μm deep. A washing solution of 5% (*w*/*v*) Triton X-100 and 5% (*v*/*v*) acetic acid was used. Prior to testing, the flow cell was cleaned sequentially with 1 mL washing solution, 1 mL isopropanol, and filtered deionized water until no particles were detected in the preview window. The sample could only be tested if the concentration of subvisible particles in the filtered water was less than or equal to 200 particles/mL. The test method was set as “flush volume 10 μL; detection volume 200 μL.” For measurement, 250 μL sample was carefully aspirated and slowly loaded into the chamber to avoid bubbles. The sample was introduced at 0.12 mL/min at room temperature, with image acquisition at 14 frames per second. Particles larger than or equal to 2 μm were recorded and analyzed.

In this study, each instrument was operated using routinely applied and internally matched combinations of flow rate, frame rate, and optical resolution as used in quality control practice; these parameters jointly determine imaging coverage and thus particle counting outcomes, and evaluating consistency under such platform-native conditions better reflects the practical objectives of standardization and regulatory calibration studies. Prior to analysis, we verified the relative consistency between the two instruments over time, which remained relatively stable, as shown in Figure S5.

### 4.3. Polystyrene Standard Microsphere Measurement

Aliquots of 500 μL of 5 μm and 10 μm polystyrene standard bead stock suspensions were each diluted to 10 mL with filtered deionized water. Subsequently, 5 mL of each suspension was further diluted 2-, 4-, 8-, and 32-fold with filtered deionized water. Dilutions were prepared under a laminar flow bench (Likang Biological Medical Technology Holdings Co., Ltd., Shanghai, China). The gradient dilutions were analyzed using both instruments under the parameters described in Section 4.2. Each sample was measured in triplicate. Subsequently, the two instruments were used to measure the same 5 μm and 10 μm polystyrene microsphere standard suspensions to verify their consistency.

### 4.4. Quantification of Particles Generated from Protein Formulations

Five randomly selected IVIG polyclonal samples, subjected to stress treatment at 25 °C with horizontal shaking at 250 rpm for 24 h, were aliquoted (6 mL each) into 7 mL vials and labeled. The aliquoting was performed under a clean bench. Both instruments, under the conditions described in Section 4.2, were used to analyze each sample in triplicate.

To prepare high particle-concentration formulations, 200 μL of IVIG stock solution (50 mg/mL) was diluted with filtered saline to 20 mL, then aliquoted into 3 mL vials (2 mL per vial). Vials were placed on an orbital shaker (Changzhou Guowang Instrument Manufacturing Co., Ltd., Changzhou, China) and agitated at 250 rpm at 25 °C for 24 h. Filtered IVIG solution (0.5 mg/mL in physiological saline) was used as the diluent, ensuring that the total protein concentration remained constant after gradient dilution. Stress-treated samples were diluted 2-, 4-, 8-, 16-, 32-, and 64-fold, labeled accordingly, and analyzed with both instruments as described in Section 4.2.

### 4.5. Detection of Mixed Particles

A volume of 1 mL of the IVIG sample, which contained elevated levels of particles prepared in Section 2.4, was thoroughly mixed with 1 mL of a 10 μm standard bead particle solution to simulate the presence of particles with different contrast in biological product samples. The preparation process was carried out in a laminar hood to avoid any particle contamination. Both instruments were used to analyze the samples according to the parameters specified in Section 4.2.

### 4.6. Clustering of Images Using Machine Learning

A convolutional neural network (CNN) was employed to learn features from raw images and perform classification [43]. ShufflenetV2 × 0.5 [44] was used as the training model, with the following parameters set: Epoch = 10, Learning rate = 0.01, Optimizer = Adam, Batch size = 2048, Image size = 16, and Random Rotate = 45, Random Horizontal Flip = 0.5, Random Vertical Flip = 0.5 for data augmentation. Of the total particle images, 70% were used as the training set, 15% as the validation set, and 15% as the test set. The Uniform Manifold Approximation and Projection (UMAP) method was used for dimensionality reduction of protein particle image features, with the following settings: dimensions = 2, Neighbor = 30, Minimum distance = 0.1, Distance metric = Euclidean, Random state seed = 2. Table S2 presents the performance comparison of different CNN architectures for subvisible particle classification, and we therefore selected the most efficient ShuffleNetV2 × 0.5 architecture.

An XGBoost [45] classification model was constructed to evaluate the functional impact of parameter differences between instruments. Particle images were randomly divided into a 70:30 training–test split. Model training was performed using an XGBoost. XGBClassifier with the following hyperparameters: n_estimators = 400, learning_rate = 0.05, max_depth = 5, subsample = 0.8, colsample_bytree = 0.8, reg_lambda = 1.0, gamma = 0.0, and eval_metric = “logloss”. The classifier was trained using all available CPU cores (n_jobs = −1) with a fixed random seed to ensure reproducibility.

### 4.7. Data Processing and Analysis

Data analyses and graphical representations were performed with GraphPad Prism version 10.1 (GraphPad Software, San Diego, CA, USA).

## 5. Conclusions

This study systematically evaluated the performance of two flow imaging microscopy (FIM) instruments from different manufacturers in both standard beads and biological samples. The results demonstrated that particle size distributions based on a unified sizing algorithm showed strong correlation and calibratability between instruments in biological sample analysis. These findings demonstrate that quantitative particle sizing and counting based on a unified algorithm exhibit cross-platform comparability, supporting the potential for data transferability for these specific metrics in the characterization of subvisible particles in injectable biopharmaceuticals. However, the persistent instrument-specific differences in image features, as revealed by machine learning analysis, indicate that achieving full image-level harmonization requires further standardization efforts. Furthermore, machine learning-driven image feature analysis validated the feasibility of cross-platform morphological recognition and classification, providing practical insights into inter-instrument data comparability.

## Figures and Tables

**Table 1:** R^2^ Values and correlation between MFA and FlowCam.

Particle Size	MFA		FlowCam	
	Equation	R^2^	Equation	R^2^
5 μm	Y = 1.003X + 2010	0.9992	Y = 0.9577X + 12583	0.9945
10 μm	Y = 1.001X − 4617	0.9857	Y = 0.9897X + 860.4	0.9887

**Table 2:** Linear regression analysis of subvisible particle counts (<25 μm) between MFA and FlowCam.

Particle Size	2–100 μm	2–5 μm	5–10 μm	10–25 μm	25–50 μm	50–100 μm
Equation	Y = 1.079X − 1230	Y = 0.7637X − 374.1	Y = 1.224X − 259.9	Y = 1.412X + 343.6	Y = 2.277X + 975.1	Y = 5.311X + 69.91
R^2^	0.9940	0.9938	0.9903	0.9936	0.9993	0.9995

**Table 3:** Confusion matrix of the machine learning model, including precision, recall, and F1 score.

	Precision	Recall	F1-Score
FlowCam-PS Particles	0.99980	0.98919	0.99447
FlowCam-Protein	0.99333	0.99988	0.99660
MFA-PS Particles	0.99990	0.99935	0.99963
MFA-Protein	0.99966	0.99995	0.99981

**Table 4:** Precision, recall, and F1 Score of the model.

	Precision	Recall	F1-Score
FlowCam-PS Particles	0.97900	0.99883	0.98881
FlowCam-Protein	0.99864	0.97582	0.98710
MFA-PS Particles	0.98027	0.91726	0.94772
MFA-Protein	0.89433	0.97432	0.93266

## Data Availability

The original contributions presented in this study are included in the article and Supplementary Materials. Further inquiries can be directed to the corresponding authors.

## Supplementary Materials

The following supporting information can be downloaded at: https://www.mdpi.com/article/10.3390/ph19010107/s1, Figure S1: Bland–Altman plots illustrating the agreement between the two instruments in detecting different particle types; Figure S2: Bland–Altman plots illustrating the agreement between the two instruments in detecting IVIG particles across different size distributions; Figure S3: Comparative dimensionality reduction analysis of particle feature distributions using (A) PCA and (B) t-SNE; Figure S4: Representative real-world images of proteinaceous particles acquired by flow imaging microscopy (FIM) instruments; Figure S5: Inter-day consistency of IVIG particle detection by two instruments; Table S1: Definition of selected particle morphology parameters [46]; Table S2: Performance comparison of different CNN architectures for subvisible particle classification.

## Abbreviations

The following abbreviations are used in this manuscript:

LO	Light Obscuration
MC	Microscopic Particle Count
FIM	Flow Imaging Microscopy
UMAP	Unified Manifold Approximation and Projection
SVPs	Subvisible particles
ChP	the Chinese Pharmacopoeia
USP	the United States Pharmacopeia
EP	the European Pharmacopoeia
NIST	National Institute of Standards and Technology
CNN	Convolutional Neural Network
ABD	Area-Based Diameter
ESD	Equivalent Spherical Diameter
