# Supplementary material for: Machine Learning-Enabled Image Comparability Assessment for Flow Imaging Microscopy Across Platforms
Source: Pharmaceuticals (Basel). 2026 Jan 7;19(1):107. doi: 10.3390/ph19010107 (PMC12845477; doi:10.3390/ph19010107)
Supplement: Supplementary file 1 [file pharmaceuticals-19-00107-s001.zip › pharmaceuticals-4058246-supplementary.pdf]

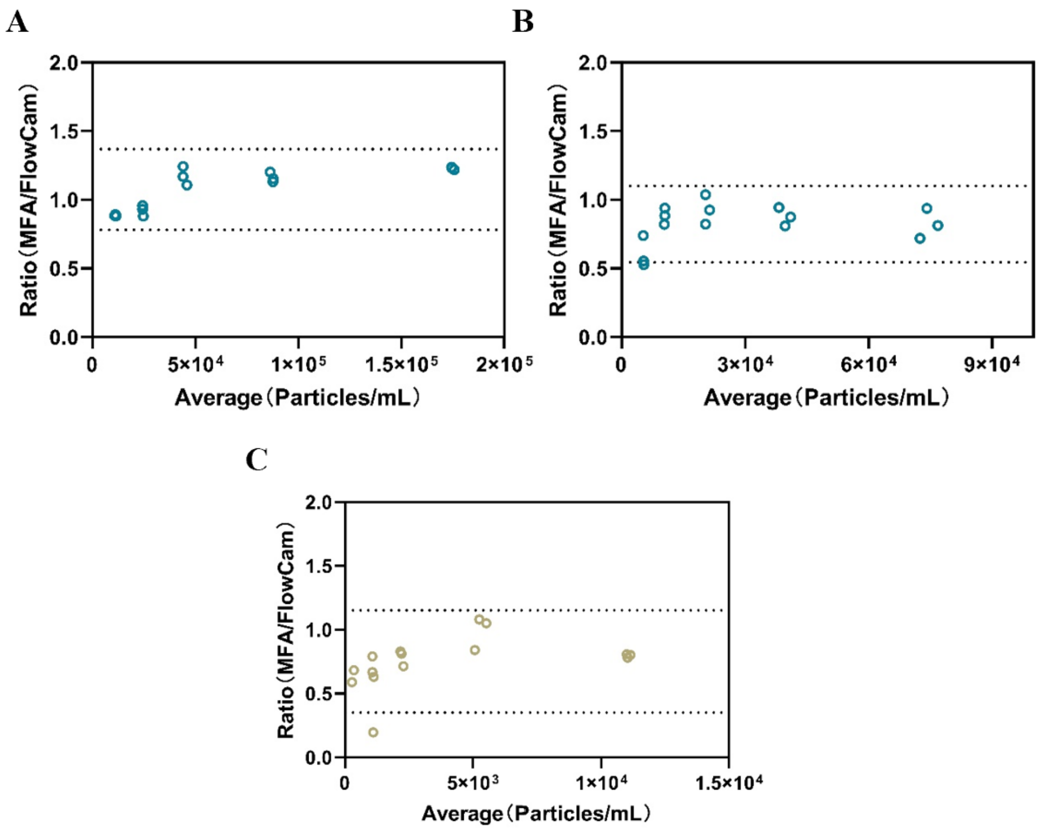


**Figure S1**. Bland-Altman plots illustrating the agreement between the two instruments in detecting different particle types. (A) 5 µm standard beads (corresponding to Figure 1B); (B) 10 µm standard beads (corresponding to Figure 1C); (C) low particle concentration IVIG sample (corresponding to Figure 2B). The dashed lines indicate the 95% agreement limits.

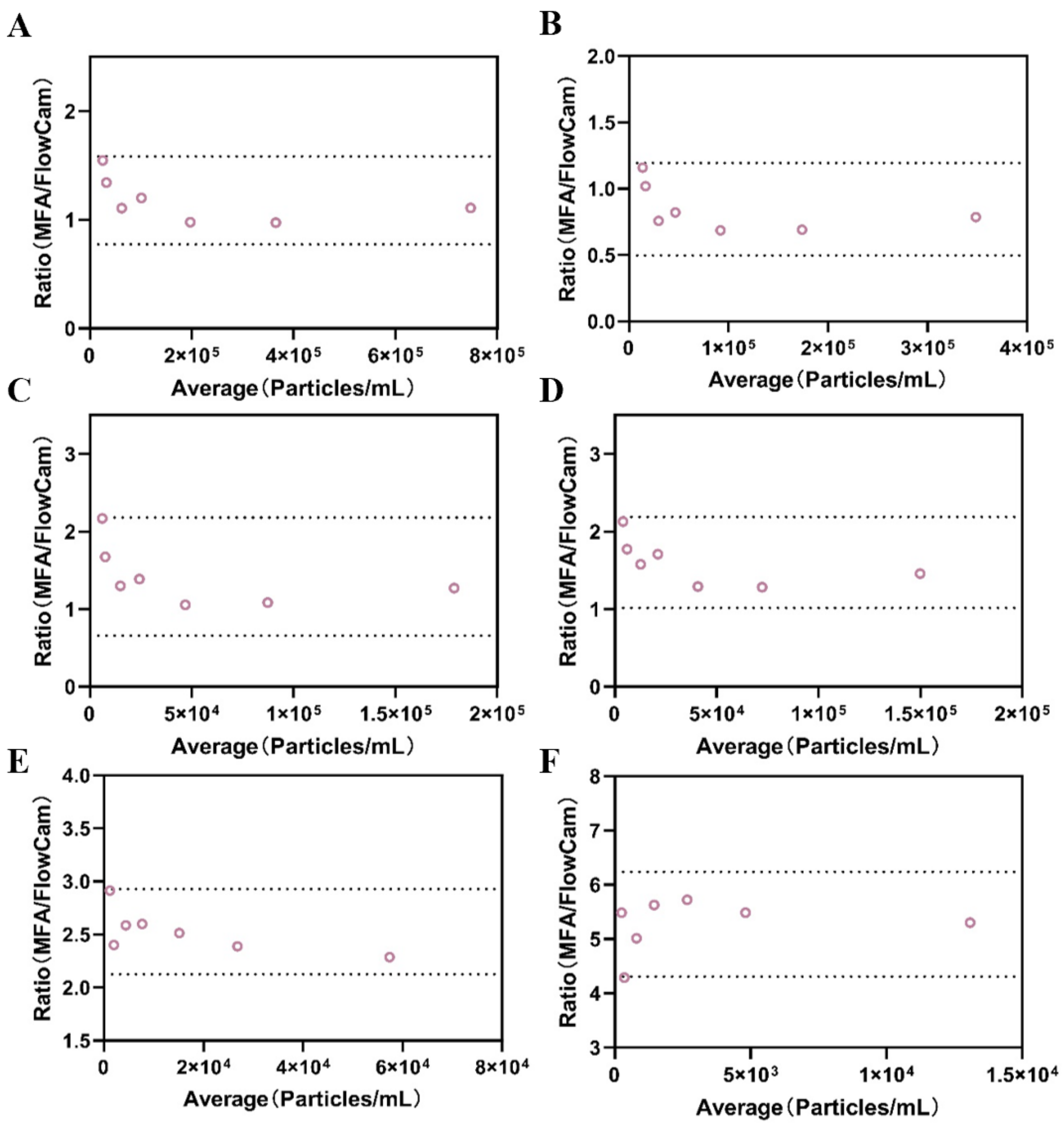


**Figure S2**. Bland-Altman plots illustrating the agreement between the two instruments in detecting IVIG particles across different size distributions (corresponding to Figure 3). (A) 2–100 µm; (B) 2–5 µm; (C) 5–10 µm; (D) 10–25 µm; (E) 25–50 µm; (F) 50–100 µm. The dashed lines indicate the 95% agreement limits.

**Table S1.** Definition of selected particle morphology parameters[1].

| Particle Morphology Parameters | Formula | Meaning |
|---|---|---|
| Compactness | $Perimeter^2/(4\pi\times Area)$ | Compactness intuitively reflects the relationship between the particle boundary and area, characterizing the degree to which the particle shape deviates from the "most compact form" (circle):<br>High compactness (large value): The particle shape is irregular, with many protrusions or is elongated, having a larger perimeter for the same area.<br>Low compactness (small value): The particle shape is close to a circle, having the shortest perimeter for the same area. |
| Circularity | $(4\pi\times Area)/Perimeter^2$ | The reciprocal of compactness |
| Aspect Ratio | Length of minor Axis/ Length of major Axis<br>Long axis: the longest diameter of the particle fitting ellipse (or the maximum FERET diameter in the 360° direction in flowcam);<br>Minor axis: the shortest diameter perpendicular to the major axis (or the shortest axis of the fitted ellipse) | Aspect Ratio quantifies the flatness or elongation of particles intuitively, revealing the size differences of particles in different directions. |
| Convexity | Actual area of particles/Area of protrusions | Convexity intuitively reflects the smoothness and integrity of the particle edges, and is particularly sensitive to detecting surface depressions or internal holes on the particle surface. |
| Area (ABD) | $2\times(\text{particle projection area}/\pi)^{1/2}$ | It intuitively quantifies the two-dimensional size of the particles, unaffected by the shape of the particles. It only focuses on the actual pixel area occupied by the particles, |

| | | |
|---|---|---|
| | | insensitive to the depressions or protrusions at the particle edges, and only reflects the “effective coverage area” of the particles. |
| Symmetry | (--) | A measure of symmetry about the center of a particle. If a particle is symmetrical about the center, the value of symmetry is 1.0. Usually used to locate (broken or partially) particles |
| Roughness | Perimeter/Convex Hull Perimeter | The core reflects the difference between the actual edge of the particle and the smooth contour (convex hull edge)- Value =1.0: the particle edge is completely smooth, without any slight bulge, burr or irregular fluctuation- Value >1.0: there are fine rough structures (such as burrs, folds, and small bulges) on the edge of particles. The higher the value, the higher the roughness of the edge The convexity focuses on the overall depression of particles (macroscopic defects), and the roughness focuses on the slight fluctuations of edges (microscopic irregularities) |
| Intensity | Grayscale sum/Number of pixels making up the particle | It directly reflects the absorption or transmission characteristics of the particles to the light source, and is a comprehensive optical manifestation of the particle's transparency, density and material. |
| Diameter (ESD) | FERET diameters in 36 uniformly distributed directions of particles were measured | A single numerical value is used to quantify the overall size of the particles, while reflecting their three-dimensional volume characteristics, regardless of the |

| | | |
|---|---|---|
| | | irregularity of the particle shape. ESD = 0: No particles exist (theoretical value); ESD > 0: Particles exist, and the larger the value, the larger the particle volume; ESD = D: Indicates that this particle has the same volume as a perfect sphere with a diameter of D. |
| Edge Gradient | $(1/N) \times \sum((Gxi2 + Gyi2)^{½})$ (i=1~N，N is the total number of particle boundary pixels)<br>Gx:Gradient component in horizontal direction (x axis)<br>Gy:Gradient component in the vertical direction (Y axis) | The intensity of brightness change between the edge of particles and the background reflects the “clarity” of particle boundaries. |
| Elongation | Length of major Axis/ Length of minor Axis<br>Long axis: the longest diameter of the particle fitting ellipse (or the maximum FERET diameter in the 360° direction in flowcam);<br>Minor axis: the shortest diameter perpendicular to the major axis (or the shortest axis of the fitted ellipse) | Elongation intuitively reflects the “elongated degree” of particles deviating from the spherical shape. The larger the value is, the more elongated the particles are and the stronger the directionality is. |
| Diameter (ABD) | $(4\times A/\pi)^{½}$<br>A: Projected area of particles (pixel area × square of pixel size) | The “two-dimensional size” of particles is visually characterized, providing a unified scale to normalize particles of arbitrary shape to equivalent circles. |

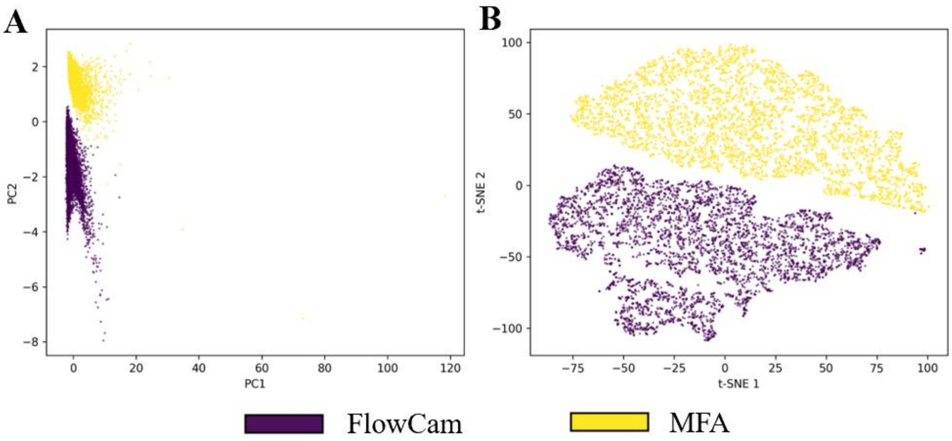


**Figure S3**. Comparative dimensionality reduction analysis of particle feature distributions using (A) PCA and (B) t-SNE.

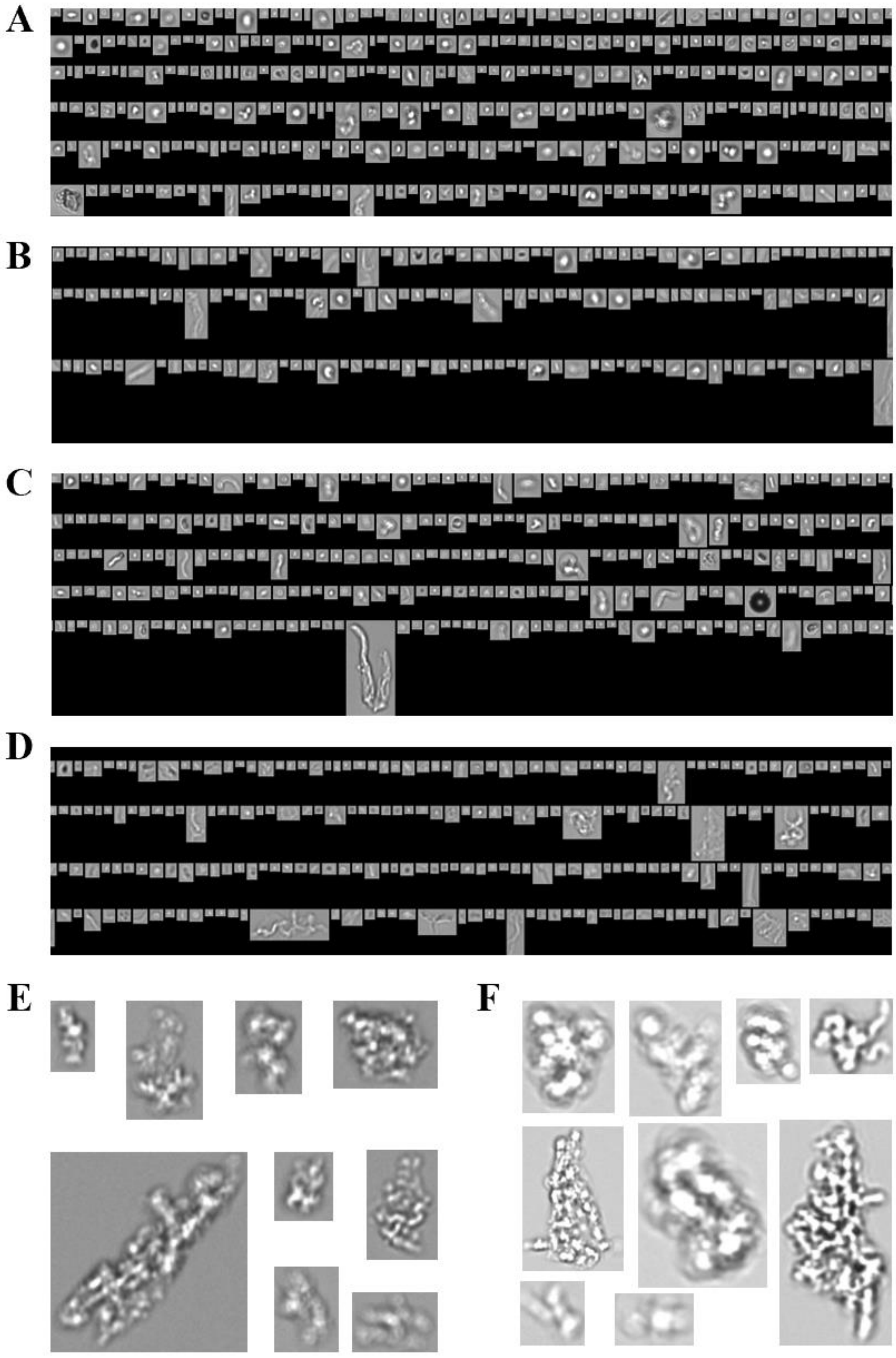


**Figure S4.** Representative real-world images of proteinaceous particles acquired by flow imaging microscopy (FIM) instruments. (A) A commercially available chimeric monoclonal antibody imaged by FlowCam; (B) A commercially available fully human monoclonal antibody imaged by FlowCam; (C) A commercially available antibody-drug conjugate (ADC) imaged by FlowCam; (D) IVIG particles imaged by FlowCam; (E) A single IVIG particle image acquired by FlowCam; (F) A single IVIG particle image acquired by MFA.

**Table S2.** Performance comparison of different CNN architectures for subvisible particle classification

| Model | Accuracy/% | Parameters/M | FLOPS/G |
|---|---|---|---|
| ResNet18 | 95.66 | 11.7 | 1.8 |
| MobileNetV3 Small | 93.20 | 2.5 | 0.06 |
| ShuffleNetV2 ×0.5 | 99.75 | 1.3 | 0.04 |

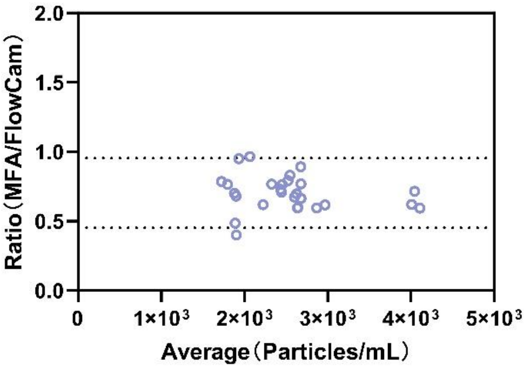


**Figure S5**. Inter-day consistency of IVIG particle detection by two instruments. Three aliquots of IVIG solutions with varying particle concentrations were randomly selected and analyzed at the same time point on Days 1, 2, and 3 using both instruments. Each sample was measured in triplicate. The particle concentrations (larger than 2 μm) were determined and are presented in this figure. The dashed lines indicate the 95% agreement limits.

## Reference


[1] H. Nishiumi *et al.*, "Utility of Three Flow Imaging Microscopy Instruments for Image Analysis in Evaluating four Types of Subvisible Particle in Biopharmaceuticals," *Journal of Pharmaceutical Sciences*, vol. 111, no. 11, pp. 3017–3028, Nov. 2022, doi: 10.1016/j.xphs.2022.08.006.
